# Influence of Exogenous Salicylic Acid and Nitric Oxide on Growth, Photosynthesis, and Ascorbate-Glutathione Cycle in Salt Stressed *Vigna angularis*

**DOI:** 10.3390/biom10010042

**Published:** 2019-12-26

**Authors:** Mohammad Abass Ahanger, Usman Aziz, Abdulaziz Abdullah Alsahli, Mohammed Nasser Alyemeni, Parvaiz Ahmad

**Affiliations:** 1 College of Life Sciences, Northwest A&F University Yangling, Xianyang 712100, Shaanxi, China; ahangerma@gmail.com; 2 College of Agronomy, Northwest A&F University Yangling, Xianyang 712100, Shaanxi, China; usmanaziz@gmail.com; 3 Botany and Microbiology Department, College of Science, King Saudi University, P.O. Box. 2460, Riyadh 11451, Saudi Arabia; aalshenaifi@ksu.edu.sa (A.A.A.); mnalyemeni@gmail.com (M.N.A.); 4 Department of Botany, S.P. College, Srinagar, Jammu and Kashmir 190001, India

**Keywords:** antioxidants, lipid peroxidation, osmolytes, salicylic acid, nitric oxide, *Vigna angularis*

## Abstract

The present study was carried out to investigate the beneficial role of exogenous application of salicylic acid (1 mM SA) and nitric oxide (100 μM NO) in preventing the oxidative damage in *Vigna angularis* triggered by salinity stress. Salinity (100 mM NaCl) stress reduced growth, biomass accumulation, chlorophyll synthesis, photosynthesis, gas exchange parameters, and photochemical efficiency (Fv/Fm) significantly. Exogenous application of SA and NO was affective in enhancing these growth and photosynthetic parameters. Salinity stress reduced relative water content over control. Further, the application of SA and NO enhanced the synthesis of proline, glycine betaine, and sugars as compared to the control as well as NaCl treated plants contributing to the maintenance of tissue water content. Exogenous application of SA and NO resulted in up-regulation of the antioxidant system. Activities of enzymatic antioxidants including superoxide dismutase (SOD), catalase (CAT), ascorbate peroxidase (APX), dehydroascorbate reductase (DHAR), and glutathione reductase (GR), as well as the content of non-enzymatic components, were more in SA + NO treated seedlings as compared to control and salinity stressed counterparts resulting in significant alleviation of the NaCl mediated oxidative damage. Content of nitrogen, potassium, and calcium increased due to SA and NO under normal conditions and NaCl stress conditions while as Na and Cl content reduced significantly.

## 1. Introduction

Salinity stress is considered one of the devastating abiotic stress factors affecting the growth and productivity of crop plants [1,2]. Excess availability of toxic salts including sodium, carbonates, etc., reduces plant growth by imparting osmotic and ionic stress [3]. It has been reported that salinity stress declines root growth, water and mineral uptake, enzyme activity, and assimilation of minerals [4]. Among the key salinity-sensitive metabolic pathways are photosynthesis, respiration, mineral assimilation, biomass accumulation, and yield productivity [5,6]. Stress-induced damaging effects are initiated by the excess accumulation of toxic free radicals commonly referred to as reactive oxygen species (ROS), which includes hydrogen peroxide, superoxide, and hydroxyl radicals [4,5]. These radicals are continuously produced at different sites within the cells. Plants are equipped with different tolerance mechanisms to counteract the damaging effects of salinity stress, which include antioxidant systems, efficient ion compartmentation, and improved accumulation of osmolytes [1,4]. It has been reported that salinity stressed plants exhibit up-regulation of antioxidant systems and osmolyte accumulation [5,7]. Nowadays, for strengthening the indigenously existing tolerance mechanisms, different strategies are being tested and adopted including conventional and biotechnological approaches [3]. The last few years have focused on the exogenous use of phytohormones for strengthening the tolerance mechanisms thus as to improve the growth and yield performance of plants.

Salicylic acid (SA) is a phenolic compound and has been reported to be involved in growth and developmental regulation of plants. As a phytohormone, SA vitally regulates growth and multiple developmental events like photosynthesis, uptake, and assimilation of essential mineral ions, enzyme activity, and stress tolerance [8,9,10]. The role of SA in improving the resistance against environmental stresses is well reported [8,11]. SA strengthens the plant immunity against toxic metals [12]. SA strengthens the stress withstanding potential by improving antioxidant functioning and glycine betaine accumulation, resulting in the protection of photosynthesis [13]. Nitric oxide is another important signaling molecule that has been reported to regulate growth and developmental events from germination to ripening [14]. However, the effects of NO can be beneficial as well as damaging depending on its concentration. Reports discussing its beneficial impact on the regulation of growth and stress tolerance in crop plants have witnessed concentration-dependent effects [15]. It has been reported that the optimal application of NO mitigates the damaging effects of salinity [16] and cadmium [17] stress by up-regulating the stress withstanding mechanisms, including antioxidant systems and osmolyte accumulation. Such beneficial effects of NO contribute significantly to the maintenance of growth by preventing damage to major cellular processes including photosynthesis. Both SA and NO are believed to crosstalk with other key phytohormones to fine tune the developmental processes and responses to stresses [11,14]. For example, Ahmad, et al. [7] have demonstrated beneficial interaction of NO with jasmonic acid reflecting in greater salinity tolerance in *Solanum lycopersicum*. However, reports discussing investigation of possible interaction, whether beneficial or damaging, between NO and SA are not available. Therefore, the present study was undertaken to understand this.

*Vigna angularis* is one of the important legume crops grown widely for its food value. It is rich in proteins, vitamins, and minerals. Its growth and productivity are affected by the biotic and abiotic stress factors like pests, fungal infection, drought, salinity etc. Therefore, the present study was carried to evaluate the role of exogenous application of SA and NO in preventing salinity stress-induced oxidative damage and growth reduction.

## 2. Materials and Methods

Healthy seeds of *Vigna angularis* were surface sterilized using 5% NaOCl for 5 minutes followed by washing with distilled water. Seeds were sown in pots filled with sand, soil, and compost in a 2:1:1 ratio. At the time of sowing, pots were irrigated with 200 mL full-strength Hoagland solution. After germination, 2 seedlings per pots were maintained and allowed to grow for 10 days. Thereafter, pots were divided into 2 groups, and to 1 group salinity stress was started by adding 100 mM NaCl to the Hoagland solution (150 mL), while the other group was maintained as normal and was irrigated with an equal amount of normal Hoagland solution. Application of SA (1 mM) and NO (100 μM, sodium nitroprusside) or SA + NO was done by a manual sprayer onto the foliage. Both salinity and SA and/ or NO treatment extended for another 20 days. Thirty-day-old seedlings were analyzed for different parameters, including oxidative stress measurement, photosynthesis, antioxidants, and osmolytes.

### 2.1. Plant Height and Dry Weight

Shoot height was measured manually, while dry weight was recorded after drying the samples in an oven for 48 h at 70 °C.

### 2.2. Estimation of Photosynthetic Pigments, Photosynthesis, and Relative Water Content

Chlorophyll and carotenoids were estimated by macerating fresh leaf tissues in acetone and the optical density of the supernatant was measured at 480, 645, and 663 nm [18] using a spectrophotometer.

Net photosynthetic rate (*Pn*), intercellular CO_2_ concentration (*Ci*), stomatal conductance (gs), and transpiration rate (*E*) were measured in fully expanded leaves using the portable photosynthetic system. For measurement of maximum photochemical efficiency (Fv/Fm), leaves were dark-adapted for 25 minutes, and measurements were carried out using Chlorophyll Fluorometer (PAM 2500; Walz, Germany).

RWC was estimated following Smart and Bingham [19], and the following formula was used for the calculation:

(1) $$\mathrm{RWC}=\frac{\mathrm{Fresh}\;\mathrm{weight}-\mathrm{Dry}\;\mathrm{weight}}{\mathrm{Turgid}\;\mathrm{weight}-\mathrm{Dry}\;\mathrm{weight}}\times 100$$

### 2.3. Estimation of Electrolyte Leakage, Hydrogen Peroxide, and Superoxide

The method described by Dionisio-Sese and Tobita [20] was used, with electrolyte leakage determination and the following formula was used for calculation:

Percent electrolyte leakage = (EC1 − EC0) / (EC2 − EC0) × 100(2)

For measurement of hydrogen peroxide (H_2_O_2_), the method described by Velikova et al. [21] was adopted and absorbance was measured at 390 nm. Concentration of H_2_O_2_ was calculated from the standard curve. Estimation of superoxide (O_2_^−^) concentrations was done following Yang et al. [22] and optical density was taken at 530 nm. For the calculation, the standard curve of NaNO_2_ was used.

### 2.4. Measurement of Lipid Peroxidation and Lipoxygenase Activity

For measuring lipid peroxidation formation of malonaldehyde content was estimated in line with the method of Madhava Rao and Sresty [23] and the extinction coefficient of 155 mM^−1^cm^−1^ was used for calculation. Lipoxygenase (LOX; EC, 1.13.11.12) activity in fresh leaf tissues was measured by monitoring the change in absorbance at 234 nm using linoleic acid as a substrate [24]. For calculation, the extinction coefficient of 25 mM^−1^ cm^−1^ was used.

### 2.5. Estimation of Proline, Glycine Betaine, and Sugars

Proline was extracted in sulphosalicylic acid according to the method of Bates et al. [25] and the absorbance was taken at 520 nm. Glycine betaine content was estimated following the Grieve and Grattan [26] method. Formation of periodide crystals with cold KI–I_2_ reagent were read at 365 nm and the standard curve of glycine betaine was used for calculation. Sugar content was estimated using the anthrone method described by Ahanger et al. [27]. After extraction in ethanol, supernatant was mixed with anthrone reagent and absorbance recorded at 620 nm.

### 2.6. Assay of Antioxidant Enzymes

Fresh 500 mg leaf tissue was macerated in ice-cold potassium phosphate buffer (100 mM, pH 7.0) supplemented with 1% PVP and 1 mM EDTA using a pre-chilled mortar and pestle. After centrifugation for 15 min at 12,000× *g* at 4 °C, supernatant was collected and used as enzyme source.

Activity of superoxide dismutase (SOD, EC1.15.1.1) was measured by estimating the ability of the enzyme (100 µL) to inhibit photochemical reduction of nitroblue tetrazolium (NBT) in an assay mixture containing 100 mM phosphate buffer (pH 7.4), 1.0 mM EDTA, 10 mM methionine, 50 µM riboflavin, and 75 µM NBT incubated under fluorescent light for 15 min. Optical density was measured spectrophotometrically at 560 nm and activity was expressed as EU mg^−1^ protein [28].

For assaying catalase (CAT, EC1.11.1.6) activity disappearance of H_2_O_2_ was recorded at 240 nm for 2 min in an assay mixture containing 50 mM phosphate buffer (pH 6.0), 0.1 mM EDTA, H_2_O_2_ and 100 µL enzyme extract in a final volume of 2 mL. Extinction coefficient of 39.4 mM^−1^cm^−1^ was used for the calculation [29].

Ascorbate peroxidase (APX, EC1.11.1.1) was assayed in an assay mixture containing 50 mM phosphate buffer (pH 7.5), 100 µL of each EDTA, ascorbate, enzyme, and H_2_O_2_. Change in absorbance was recorded at 290 nm for 2 min and the extinction coefficient of 2.8 mM^−1^cm^−1^ was used for calculation [30].

Activity of glutathione reductase (GR, EC1.6.4.2) was assayed by measuring the change in absorbance at 340 nm for 3 min in a reaction mixture containing 100 mM potassium phosphate buffer (pH 7.0), EDTA, 50 μM NADPH, 100 μM oxidized glutathione, and 100 μL enzyme [31].

Dehydroascorbate reductase (DHAR, EC: 1.8.5.1) activity was assayed by monitoring change in absorbance at 265 nm and extinction coefficient of 14 mM^−1^ cm^−1^ was used for the calculation [30].

### 2.7. Estimation of Ascorbate, Reduced Glutathione, and Tocopherol

For estimation of ascorbate (AsA) content, fresh leaf material was extracted in 6% TCA and supernatant was reacted with dinitrophenylhydrazine (2%) and thiourea (10%). Absorbance was taken at 530 nm and calculations were done using the standard curve of AsA [32]. For estimation of reduced glutathione (GSH), fresh tissue was homogenized in phosphate buffer (pH 8.0) and 500 µL supernatant was reacted with 5,5-dithiobis-2-nitrobenzoic acid. Optical density was taken at 412 nm and concentration of GSH was determined from the standard graph of GSH [33]. For estimation of tocopherol tissue was extracted in ethanol and petroleum ether (1.6:2 and supernatant was incubated with 2% of 2,2-dipyridyl in dark. Absorbance was recorded at 520 nm and the standard curve was used for calculation [34].

### 2.8. Estimation of N, K, Ca, Na, and Cl

K, Na, and Ca were estimated flame photometrically described by Ahanger et al. [27]. Micro-Kzeldahl’s method suggested by Jackson [35] was employed for estimation of N content. Chloride was estimated by titrating the extract against AgNO_3_ using K_2_CrO_4_ as an indicator.

### 2.9. Statistical Analysis

The mean (±SE) of 4 replicates were presented. Statistical analysis for a completely randomized design was carried using the analysis of variance and the least significant difference (LSD) was calculated at *p* < 0.05.

## 3. Results

Salinity stress declined the growth of *Vigna angularis* significantly over the control, and the application of NO or SA, individually or in combination, mitigated the decline to considerable levels. Relative to the control, height and weight declined by 30.45% and 41.37% due to salinity stress. Height and dry weight increased by 31.85% and 39.89% respectively in NaCl + SA + NO application over the NaCl stressed plants (Table 1).

**Figure 1:**
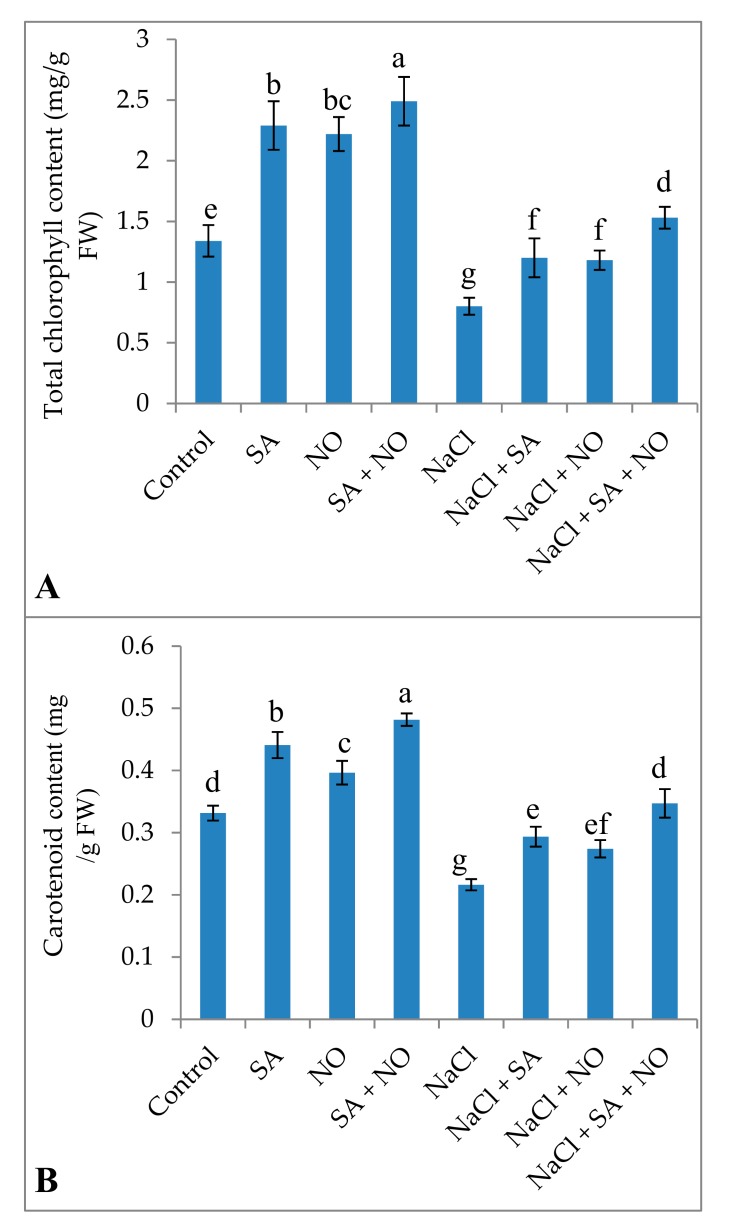
Effect of exogenous application of SA and NO on (**A**) total chlorophyll and (**B**) carotenoids in *Vigna angularis* under salinity (100 mM NaCl) stress. Data are the mean of four replicates and different letters show significant difference at *p* < 0.05.

**Figure 2:**
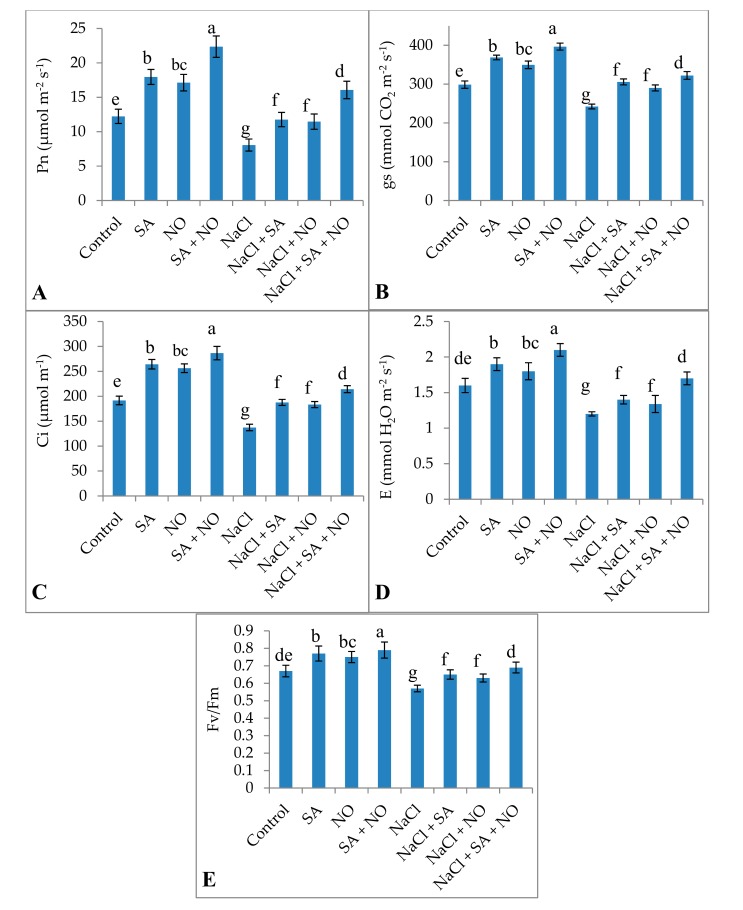
Effect of exogenous application of SA and NO on (**A**) net photosynthesis (*Pn*), (**B**) stomatal conductance (*gs*), (**C**) intercellular CO_2_ (*Ci*), (**D**) transpiration (*E*) and (**E**) PSII activity (Fv/Fm) in *Vigna angularis* under salinity (100 mM NaCl) stress. Data are the mean of four replicates and different letters show significant difference at *p* < 0.05.

Exogenous application of SA or NO and SA + NO improved the total chlorophyll and carotenoid content, gas exchange attributes including net photosynthesis, stomatal conductance, transpiration rate, and intercellular CO_2_ significantly over the control. Maximal enhancement in total chlorophylls (46.18%), carotenoids (31.18%), Pn (45.30%), gs (24.71%), E (23.80%), Ci (33.40%), and Fv/Fm (15.18%) was observed in seedlings treated with SA + NO. However, salinity stress resulted in the decline of 40.22% for chlorophylls, 34.75% for carotenoids, 34.09% for Pn, 18.85% for gs, 25.05% for E, 28.34% for Ci, and 14.92% for Fv/Fm. Salinity stress declined these parameters significantly over the control and application of NO and /or SA ameliorated the decline maximally when applied combinedly. Relative to NaCl stressed plants total chlorophylls, carotenoids, Pn, gs, E, Ci and Fv/Fm increased by 47.64%, 37.68%, 49.81%, 24.82%, 29.41%, 35.93%, and 17.39% respectively in NaCl + SA + NO treated seedlings (Figure 1 and Figure 2).

**Figure 3:**
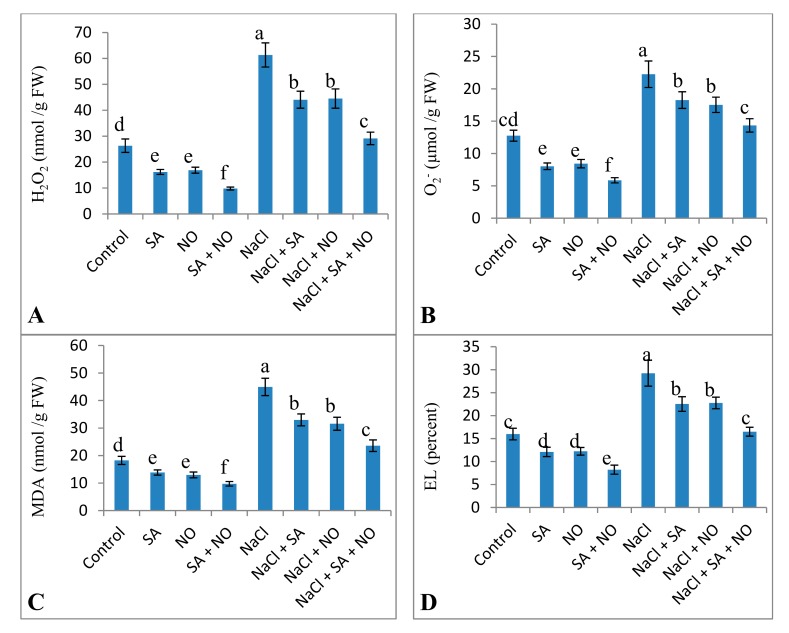
Effect of exogenous application of SA and NO on (**A**) hydrogen peroxide, (**B**) superoxide, (**C**) lipid peroxidation (MDA), and (**D**) electrolyte leakage (EL) in *Vigna angularis* under salinity (100 mM NaCl) stress. Data are the mean of four replicates and different letters show significant difference at *p*< 0.05.

**Figure 4:**
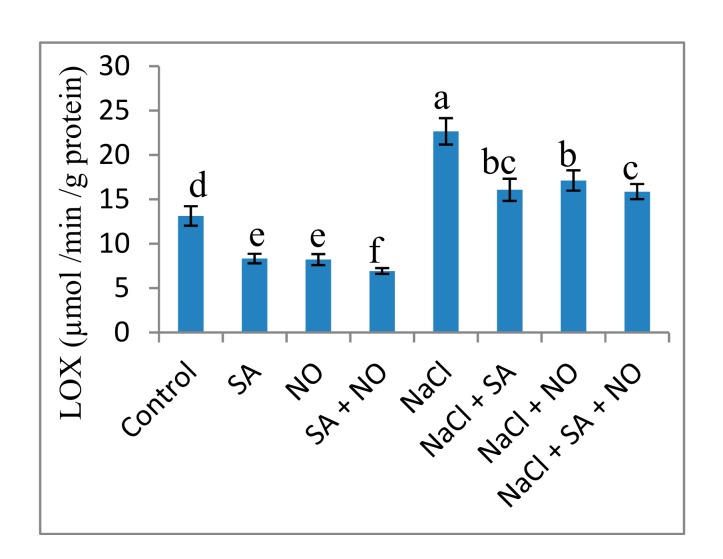
Effect of exogenous application of SA and NO on activity of lipoxygenase in *Vigna angularis* under salinity (100 mM NaCl) stress. Data are the mean of four replicates and different letters show significant difference at *p*< 0.05.

Exposure to salinity stress resulted in an increase in the oxidative parameters studied. Relative to the control, NaCl treatment increased H_2_O_2_ (57.04%), O_2_^−^ (42.67%), MDA (58.45%), EL (45.33%) and LOX (42.05%) activity. Foliar application of NO and SA decreased these parameters significantly with maximal decline of 62.82%, 54.07%, 46.62%, 48.49%, and 47.29% in H_2_O_2_, O_2_^−^, MDA, EL and LOX activity respectively over the control. Application of NO and/or SA to NaCl stressed seedlings declined these parameters with a maximal decline of 52.47% for H_2_O_2_, 34.96% for O_2_^−^, 47.55% for MDA, 43.55% for EL and 29.96% for LOX activity attained with NaCl + SA + NO over the NaCl stressed plants (Figure 3 and Figure 4).

*Vigna angularis* exposed to salinity stress exhibited increased accumulation of proline (21.92%), sugars (14.93%) and glycine betaine (23.49%) content over the control. Under normal growth conditions, supplementation of SA and NO individually increased the content of proline, sugars, and glycine betaine with maximal increase observed due to their combined application. When SA and /or NO were supplied to NaCl stressed seedlings content of proline, sugars, and glycine betaine exhibited a further increase of 37.61%, 41.89%, and 51.08% respectively over the NaCl stressed plants (Table 2). Salinity declined RWC by 23.02% over the control and application of SA + NO ameliorated the decline by 22.34% over NaCl stressed seedlings (Table 2).

**Figure 5:**
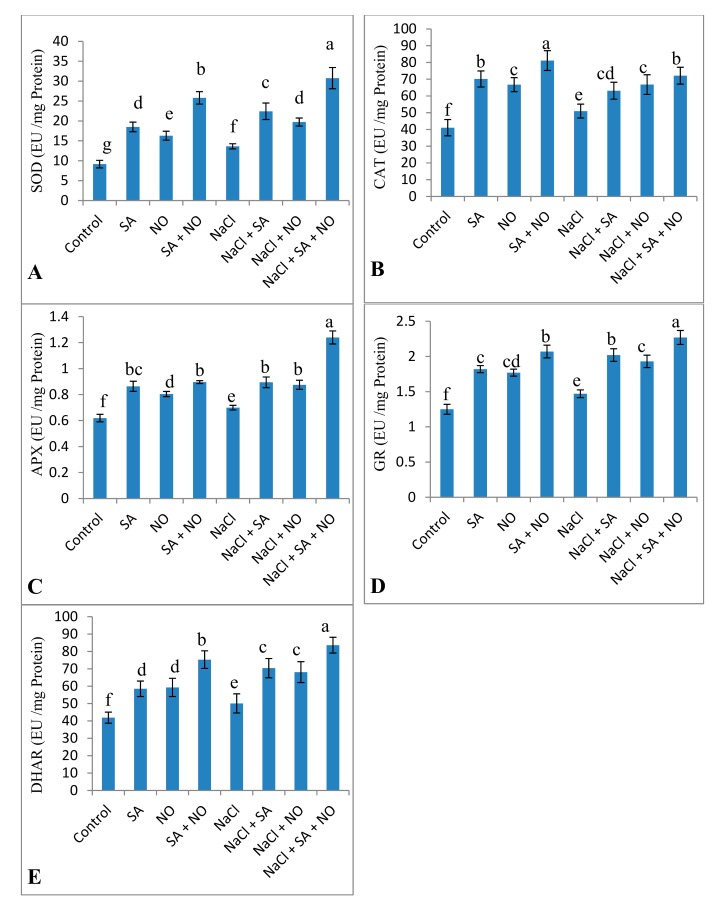
Effect of exogenous application of SA and NO on activity of (**A**) superoxide dismutase (SOD), (**B**) catalase (CAT), (**C**) ascorbate peroxidase (APX), (**D**) glutathione reductase (GR) and (**E**) DHAR in *Vigna angularis* under salinity (100 mM NaCl) stress. Data are the mean of four replicates and different letters show significant difference at *p* < 0.05.

**Figure 6:**
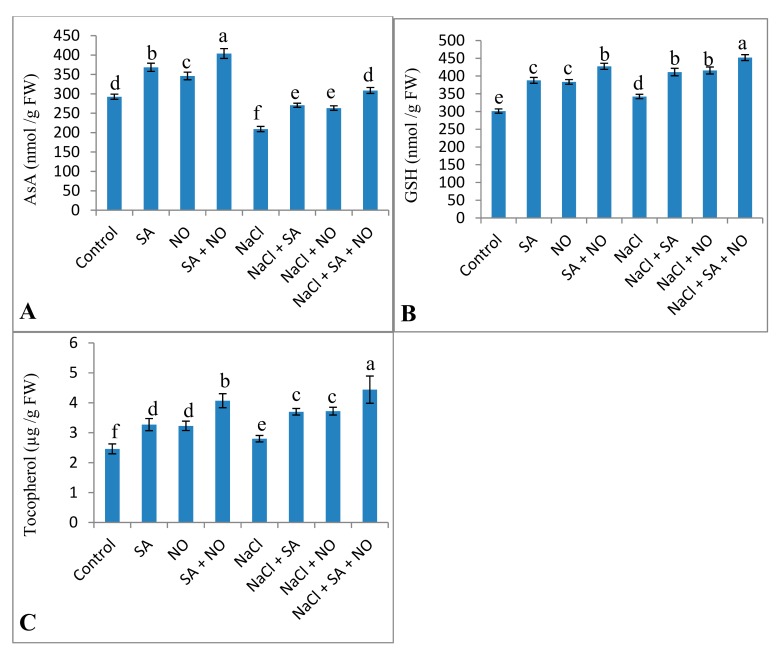
Effect of exogenous application of SA and NO on (**A**) ascorbic acid, (**B**) reduced glutathione and (**C**) tocopherol content in *Vigna angularis* under salinity (100 mM NaCl) stress. Data are the mean of four replicates and different letters show significant difference at *p* < 0.05.

Activities of antioxidant enzymes, including SOD, CAT, APX, DHAR, and GR, increased due to the application of NO and SA under normal and salinity stress conditions. Percent increase was 32.79% for SOD, 19.42% for CAT, 11.59% for APX, 14.96% for GR, and 16.36% for DHAR due to NaCl stress, however, this was further increased by 55.68%, 29.32%, 43.58%, 35.24%, and 40.07% respectively due to combined application of SA + NO over the NaCl stressed ones (Figure 5). Salinity decreased AsA by 28.47% but increased the content of GSH and tocopherol by 11.98% and 12.14% over the control. Foliar spray of SA and NO resulted in increased AsA, GSH and tocopherol content under normal as well as salinity stressed conditions. GSH and tocopherol maximally increased by 33.32% and 44.59% in NaCl + SA + NO treated seedlings. Decline in AsA was mitigated by application of SA and NO with maximal mitigation of 32.20% in NaCl + SA+ NO treated seedlings (Figure 6).

Content of Na and Cl increased due to NaCl stress and application of SA and NO declined their accumulation significantly. Relative to control, Na and Cl increased by 61.05% and 68.78% due to NaCl treatment, however, the application of SA + NO reduced the content of Na and Cl by 50.07% and 47.77% over the NaCl stressed plants. N, K and Ca declined by 40.10%, 45.07%, and 33.33% due to NaCl treatment over the control. Application of SA and NO increased the content of N, K, and Ca significantly over the control with a maximal increase of 29.96%, 27.91%, and 50.58% in SA + NO treated seedlings. Relative to NaCl stressed plants, decline in N, K and Ca was ameliorated by 38.12, 43.88 and 33.33% in NaCl + SA + NO treated plants (Table 3).

## 4. Discussion

In the contemporary era, a cumbersome challenge for plant scientists and agriculturalists has been to develop strategies to improve the stress tolerance potential in plants. Such beneficial strategies result in the protection of global food security [36]. In the present study, the role of exogenous supplementation of NO and SA individually or combinedly was studied, and it was observed that the combined application of SA and NO proved much more beneficial in ameliorating the growth decline under salinity stress. Considerable research reports have described the salinity mediated growth decline in terms of height and biomass accumulation [5,7,8]. The application of SA [37] and NO [7] have been reported to improve the growth and biomass accumulation in salinity stressed *Brassica juncea* and chickpea, respectively. Reduced growth and biomass accumulation under salinity stress is attributed to the osmotic and ionic effects resulting in decline cellular division and proliferation [38]. SA [11] and NO [39] are believed to interact with other important molecules to protect growth retardations under stress. In the present study combined application of SA and NO proved much more beneficial in mitigating the decline in growth and biomass accumulation. Further, increased growth under salinity stress due to SA and/or NO application was correlated with their positive influence on the photosynthetic parameters including gas exchange parameters and PSII functioning. Earlier salinity mediated reduction in chlorophyll synthesis [5] and photosynthesis [13] has been reported in wheat and mungbean, respectively. Reduced chlorophyll content due to salinity results from its effect on the uptake of magnesium and nitrogen [40]. Moreover, stresses trigger greater damage to chlorophyll by up-regulating chlorophyllase activity and down-regulating the enzymes mediating chlorophyll synthesis [41]. Exogenous SA and NO have been reported to improve photosynthetic functioning under salinity stress by enhancing chlorophyll synthesis and improving gas exchange [13,17]. Reduced mineral uptake under salinity directly influences photosynthetic functioning and NO application improves mineral uptake significantly [40]. Improved gas exchange and PSII activity in SA and NO treated seedlings determines their role in photosynthetic control and protection through their involvement in stomatal and non-stomatal attributes. Interaction of NO with JA [7] has been reported to increase chlorophyll synthesis under salinity stress. Exogenous application of SA has been reported to prevent salinity induced photosynthetic arrest in *Brassica juncea* [37]. From a recent study, it can be concluded that improved photosynthesis due to SA and NO application may have resulted due to up-regulation of antioxidant and osmolyte accumulation leading to lesser ROS accumulation and maintenance of tissue water content, respectively [16,42,43,44,45].

Greater generation of ROS, including H_2_O_2_ and O_2_ resulted due to salinity stress, has earlier been reported by several workers [16,46,47]. Reduced accumulation of ROS due to NO [16] and SA [37] has been reported. Recently, Ahanger et al. [6] have demonstrated that reduced ROS accumulation and lipoxygenase activity reflected in improved membrane functioning and growth performance under salinity stress. Reduction in oxidative damage under stress due to NO application has been reported due to its effects on the accumulation of H_2_O_2_ and O_2_ as well as reduced activity of lipoxygenase [48]. Excess accumulation of toxic radicals like H_2_O_2_ or O_2_ drastically affected the membrane functioning by initiating peroxidation of lipids and lessening their generation due to exogenous application of SA and /or NO could benefit plants to maximize the performance under stress conditions [8,13,17]. In corroboration with our results, reduced oxidative effects due exogenous application of SA [1,37] and NO [7,16,17] have been earlier reported, however, reports discussing their interactive role are scanty. Reduced accumulation of ROS in SA and NO treated seedlings may have directly contributed to the protection of key cellular organelles like chloroplast, mitochondria, thereby allowing their smooth functioning.

It was interesting to note that reduced oxidative damage in SA and NO treated seedlings was correlated with significant up-regulation of the antioxidant system in them. Plants potentially improve the antioxidant functioning to counter the deleterious effects of stress [43,45,46]. Antioxidant system constituted of enzymatic and non-enzymatic components operates in cells neutralize ROS to maintain their levels below toxic concentrations [7,17,49]. Up-regulation of the antioxidant system in repose to salinity stress has been reported earlier in mustard [8], wheat [5], and pepper [40]. Chen et al. [50] have demonstrated that an up-regulated antioxidant system due to SA application protects PSII activity in wheat. Exogenous application of NO up-regulates the activity of antioxidant enzymes in salt-stressed pepper seedlings leading to the prevention of oxidative effects of ROS on photosynthesis, mineral uptake, and assimilation. SOD acts on superoxide while H_2_O_2_ is neutralized by either CAT or APX in AsA-GSH cycle. APX, DHAR, and GR form enzymatic components of AsA-GSH cycle, while as ascorbate and glutathione form its non-enzymatic components [51]. AsA and GSH act as redox buffers besides their radical neutralizing function [51,52]. Up-regulation of the AsA-GSH cycle functioning due to exogenous application of SA [9] and NO [7,17] has been reported to prevent deleterious effects of stresses. Raman and Ravi [53] have demonstrated that the application of SA resulted in increased activities of SOD, CAT, and APX in *Haematococcus pluvialis*. Tocopherols exist in the chloroplast envelope, thylakoid membrane, and plastoglobuli, where it protects them from the damaging effects of ROS [54]. SA- and NO-mediated increase in tocopherol content contributed to strengthening of the antioxidant system thereby protecting the major cellular processes like photosynthesis, which was evident in the present study as well. Yusuf et al. [55] have demonstrated that the over-expression of tocopherol synthesis increases abiotic stress tolerance by protecting chlorophyll synthesis and chlorophyll fluorescence in *Brassica juncea*. It has been reported that GSH, SA, and ethylene interplay with each other to counteract the stress damage efficiently [56]. Improved GSH, AsA, and tocopherol content due to SA and NO application may have contributed to the regulation of such phytohormone dependent signaling networks for improved salinity tolerance. Further studies in this direction will be noteworthy.

Increased accumulation of proline, glycine betaine, and sugars due to SA and NO application was obvious in the present study. Compatible organic osmolytes prevent stress-induced damage to cellular organelles by protecting membrane structures, enzyme functioning, and maintaining water content [44,52]. Increased accumulation of proline, glycine betaine, sugars, and amino acids under salinity stress have been reported by several workers [5,8,57,58]. Plants accumulating significantly increased concentrations of osmolytes withstand the stressful conditions better [59]. Transgenic plants over-expressing proline biosynthesizing genes exhibit greater stress tolerance [60]. Greater accumulation of osmolytes like proline results from the up-regulation of their biosynthesizing pathways [4]. Proline and glycine betaine supplementation have been reported to prevent oxidative effects of salinity stress as well as lowered cell death [61]. Khan et al. [13] have demonstrated SA mediated improved salinity tolerance and photosynthetic protection in mungbean due to increased glycine betaine accumulation. Similarly, Ahmad et al. [7] have reported NO application resulting in greater accumulation of proline and glycine betaine resulting in enhanced salinity tolerance. However, reports discussing the interactive influence of SA and NO on osmolyte metabolism are rare. Increased accumulation of osmolytes like proline results from the improved mineral assimilation like N making the precursors for the synthesis of other amino acids available as well [43]. It has been proposed that improved stress in plants can be achieved by dissecting the osmolyte biosynthetic pathways [62]. Osmolytes help plants recover quickly after stress release [44].

The decline in uptake of mineral nutrients, including N, K, and Ca, was mitigated by the application of SA and/ or NO. Earlier increased mineral uptake due to SA [37,63] and NO [52,64] has been reported, however, reports discussing their interactive role in the regulation of mineral uptake are rare. Sheng et al. [65] have demonstrated increased uptake of K and Ca due to SA in wheat, resulting in the up-regulation of antioxidant system and mitigation of oxidative effects under manganese stress. Increased uptake of mineral nutrients influences the cellular functioning and whole plant performance by regulating processes like photosynthesis, antioxidant systems, and enzyme activity [42,66]. In salt-stressed cucumber, foliar application of SA has been reported to mitigate the reduction in uptake of key macro and microelements. Dong et al. [45] have also reported NO-mediated increased uptake of key mineral elements like K, Mg, Ca, and Zn, resulting in a significant enhancement in growth through modulations in the photosynthesis and antioxidant system. Therefore, it could be inferred from the present study that SA and/ or NO-mediated enhancement in the mineral uptake of *Vigna angularis* may have contributed to improved growth and salinity tolerance by regulating the major cellular processes. The content of Na and Cl was significantly declined due to SA and NO application.

## 5. Conclusions

Exogenous application of SA and NO considerably averted the deleterious effects of salinity stress in *Vigna angularis* by declining the generation of ROS and improving mineral ion uptake. The combined application of SA and NO proved more beneficial as compared to their individual application, resulting in greater protection against salinity stress. Up-regulation of the antioxidant system and increased accumulation of osmolytes in SA and NO-treated seedlings evidently contributed to improved growth and photosynthesis. The present study suggests beneficial interaction between SA and NO for growth protection under salinity stress.

## Figures and Tables

**Table 1:** Effect of exogenous application of salicylic acid (SA) and nitric oxide (NO) on shoot length (cm) and plant dry weight (g/plant) in *Vigna angularis* under salinity (100 mM NaCl) stress. Data are the mean of four replicates and different letters show a significant difference at *p* < 0.05.

Treatments	Shoot Length	Dry Weight
Control	24.3 ± 2.04c	2.03 ± 0.063c
SA	29.1 ± 2.11b	2.86 ± 0.050b
NO	27.9 ± 2.07b	2.77 ± 0.054b
SA + NO	32.0 ± 2.40a	3.23 ± 0.065a
NaCl	16.9 ± 1.14e	1.19 ± 0.033e
NaCl + SA	19.4 ± 1.08d	1.66 ± 0.040d
NaCl + NO	18.9 ± 1.62d	1.59 ± 0.049d
NaCl + SA + NO	24.8 ± 2.16c	1.98 ± 0.053c

**Table 2:** Effect of exogenous application of SA and NO on proline, glycine betaine, sugars and relative water content in *Vigna angularis* under salinity (100 mM NaCl) stress. Data are the mean of four replicates and different letters show significant difference at *p* < 0.05.

Treatments	Proline	Glycine Betaine	Sugars	RWC
Control	41.3 ± 3.64f	2.93 ± 0.32f	4.33 ± 0.45f	76.43 ± 4.10c
SA	67.9 ± 4.50c	4.86 ± 0.25d	6.26 ± 0.45d	83.23 ± 4.70b
NO	62.8 ± 4.08d	4.66 ± 0.47d	6.16 ± 0.47d	82.90 ± 5.60b
SA + NO	81.0 ± 5.50a	5.73 ± 0.50b	7.83 ± 0.60b	88.46 ± 4.07a
NaCl	52.9 ± 4.91e	3.83 ± 0.37e	5.09 ± 0.27e	58.83 ± 3.81e
NaCl + SA	83.0 ± 4.92a	5.73 ± 0.43b	7.23 ± 0.83bc	68.17 ± 4.29d
NaCl + NO	77.4 ± 5.48b	5.36 ± 0.41c	6.96 ± 0.80c	65.13 ± 3.94d
NaCl + SA + NO	84.8 ± 5.26a	7.83 ± 0.51a	8.76 ± 0.76a	75.76 ± 4.33c

**Table 3:** Effect of exogenous application of SA and NO on uptake of Na, Cl, N, K, and Ca (mg g^−1^ DW) in *Vigna angularis* under salinity (100 mM NaCl) stress. Data are the mean of four replicates and different letters show a significant difference at *p* < 0.05.

Treatments	Na	Cl	N	K	Ca
Control	5.25 ± 0.27g	3.43 ± 0.32e	18.7 ± 1.3c	14.2 ± 1.1c	4.2 ± 0.081c
SA	3.41 ± 0.20e	2.86 ± 0.19d	23.8 ± 2.1b	17.1 ± 1.6b	6.8 ± 0.085b
NO	3.50 ± 0.13e	2.82 ± 0.23d	22.5 ± 1.5b	16.8 ± 1.3b	6.3 ± 0.043b
SA + NO	3.04 ± 0.19f	2.45 ± 0.25f	26.7 ± 1.9a	19.7 ± 2.1a	8.5 ± 0.18a
NaCl	13.48 ± 1.71a	10.99 ± 1.08a	11.2 ± 0.87e	7.8 ± 0.63e	2.8 ± 0.07e
NaCl + SA	9.00 ± 0.15c	8.10 ± 0.17b	15.5 ± 1.1d	10.4 ± 0.91d	3.4 ± 0.093d
NaCl + NO	9.77 ± 1.23b	8.22 ± 0.36b	15.3 ± 1.3d	9.2 ± 0.84d	3.0 ± 0.091d
NaCl + SA + NO	6.73 ± 0.35d	5.74 ± 0.64c	18.1 ± 1.6c	13.9 ± 1.0c	4.2 ± 0.043c
